# Research on 3D Reconstruction of Binocular Vision Based on Thermal Infrared

**DOI:** 10.3390/s23177372

**Published:** 2023-08-24

**Authors:** Huaizhou Li, Shuaijun Wang, Zhenpeng Bai, Hong Wang, Sen Li, Shupei Wen

**Affiliations:** College of Building Environmental Engineering, Zhengzhou University of Light Industry, Zhengzhou 450001, China; lihuaizhou@zzuli.edu.cn (H.L.); 332122020810@email.zzuli.edu.cn (S.W.); baiyi1056@zzuli.edu.cn (Z.B.); wanghong@zzuli.edu.cn (H.W.); 332313030908@email.zzuli.edu.cn (S.W.)

**Keywords:** binocular vision, thermal infrared, stereo matching, 3D reconstruction

## Abstract

Thermal infrared imaging is less affected by lighting conditions and smoke compared to visible light imaging. However, thermal infrared images often have lower resolution and lack rich texture details, making them unsuitable for stereo matching and 3D reconstruction. To enhance the quality of infrared stereo imaging, we propose an advanced stereo matching algorithm. Firstly, the images undergo preprocessing using a non-local mean noise reduction algorithm to remove thermal noise and achieve a smoother result. Subsequently, we perform camera calibration using a custom-made chessboard calibration board and Zhang’s camera calibration method to obtain accurate camera parameters. Finally, the disparity map is generated using the SGBM (semi-global block matching) algorithm based on the weighted least squares method, enabling the 3D point cloud reconstruction of the object. The experimental results demonstrate that the proposed algorithm performs well in objects with sufficient thermal contrast and relatively simple scenes. The proposed algorithm reduces the average error value by 10.9 mm and the absolute value of the average error by 1.07% when compared with the traditional SGBM algorithm, resulting in improved stereo matching accuracy for thermal infrared imaging. While ensuring accuracy, our proposed algorithm achieves the stereo reconstruction of the object with a good visual effect, thereby holding high practical value.

## 1. Introduction

Binocular stereo vision is a prominent research focus in the field of machine vision [1]. Over the years, it has played a vital role in various domains, including 3D reconstruction [2], aerospace [3], and unmanned driving [4]. Among existing depth-sensing technologies, stereo vision stands out as the only passive method, offering advantages such as low cost, ease of implementation, and the ability to range under non-contact conditions. By employing a stereo matching algorithm, it identifies corresponding pixel pairs in the left and right views, calculates the disparity of homonymous points to extract depth information from the image, and enables three-dimensional reconstruction of the target scene [5].

The stereo matching algorithm is a crucial step in the entire process of stereo vision 3D reconstruction [6]. Commonly used stereo matching algorithms can be categorized into two types: feature-point-based matching and region-based matching [7]. Feature-point-based matching methods rely on extracting key feature points in an image and then estimating the disparity by matching these feature points between two images, resulting in a sparse reconstruction of an object. Widely used feature-point-based matching algorithms include SIFT (scale-invariant feature transform) [8], SURF (speeded up robust features) [9], and ORB (oriented FAST and rotated BRIEF) [10]. On the other hand, region-based matching methods enable dense stereo reconstruction by dividing the image into several regions or windows and estimating the disparity by comparing the similarity between these regions. Common region-based matching algorithms include local matching algorithms [11], global matching algorithms [12], and semi-global matching algorithms [13]. However, traditional region-based matching algorithms often face challenges in dealing with texture loss and edge blurring, resulting in difficulties in obtaining accurate matches [14]. This limitation negatively impacts subsequent 3D reconstruction.

Researchers have addressed the limitations of current matching algorithms by proposing improved algorithms to enhance their robustness and accuracy. Wang et al. [15] proposed an improved AD-Census algorithm that optimizes the average window pixel algorithm and overcomes the sensitivity to the center pixel value. The improved AD-Census algorithm enhances object contours in the disparity maps and significantly improves the image edges. Lu et al. [16] introduced an adaptive weighted path cost aggregation algorithm based on SGBM, which involved image downsampling and Gaussian filtering to reduce computation and enhance the accuracy and computational efficiency of the algorithm. In response to the drawbacks of the dynamic programming (DP) algorithm, Zhao et al. [17] presented a 3D reconstruction algorithm based on SGBM, augmenting photo quality through histogram equalization, minimizing the impact of external factors, and improving the matching rate of the algorithm. Moreover, Zhao et al. [18] proposed an AD-Census stereo matching algorithm based on gradient division for weak texture regions, effectively improving the matching in such regions and achieving precise localization and ranging of target points, ultimately accomplishing the basic reconstruction of the indoor target scene contour. Improved stereo matching algorithms have been widely studied and applied in the field of computer vision. However, the existing stereo matching techniques are mainly for visible light, and it will be difficult for visible light cameras to perform stereo matching and three-dimensional reconstruction when there is dense smoke or dark environments. In contrast, thermal infrared image technology has good cloud penetration ability and is not affected by light conditions. In certain complex scenarios and environments, thermal infrared technology offers significant advantages. As the development of infrared technology continues and its costs decrease, it has found applications in various fields. For instance, Zhou et al. [19] proposed IPD-Net, a solution for pedestrian detection in challenging lighting and weather conditions, which improved the accuracy of pedestrian detection in infrared images by 3.6% compared to YOLOv5s. Meanwhile, Reham et al. [20] utilized outdoor infrared thermography for capturing images of PV modules and introduced an adaptive neuro-fuzzy inference system (ANFIS) for fault classification, successfully detecting and classifying faults in PV modules. Additionally, Mauren et al. [21] proposed a method for 3D reconstruction and visualization of organs based on thermal infrared and CT images. This approach overcame the limitations of traditional 2D images by incorporating spatial depth information, thereby enhancing applications in the biomedical science field. However, despite the wide application of infrared technology in various fields, the current stereo matching algorithms for thermal infrared stereo matching and 3D reconstruction face challenges due to the low resolution and lack of texture in thermal infrared images [22]. Therefore, this study proposes a stereo matching algorithm based on SGBM to overcome these challenges in environmental surveying and thermal infrared stereo matching under low visibility conditions. The specific contributions are as follows: firstly, the development of a custom chessboard calibration board tailored specifically for thermal infrared calibration, providing accurate calibration tools for thermal infrared stereo matching. Secondly, the introduction of an effective non-local means denoising algorithm to reduce noise and improve the image quality of thermal infrared images. Thirdly, the enhancement of stereo matching accuracy in thermal infrared images through the solution of the disparity problem using a weighted least squares method. Lastly, the implementation of an optimized stereo matching algorithm to achieve 3D reconstruction of objects in thermal infrared images.

## 2. Materials and Methods

**Figure 1:**
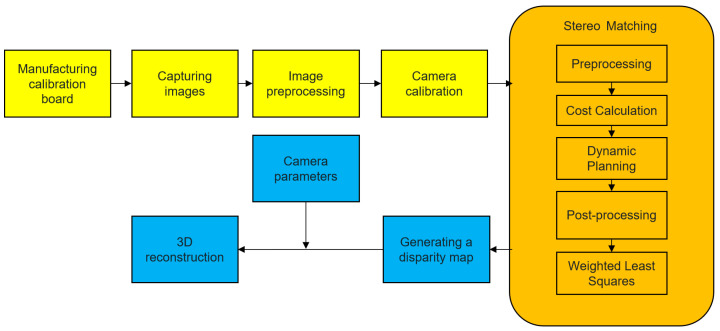
3D reconstruction system.

Figure 1 shows our proposed 3D reconstruction system. It can be summarized into three stages.

The first stage is image preprocessing and camera calibration. Firstly, we manufacture a chessboard calibration board and capture images of the calibration board. Then, these images are processed in parallel threads to remove noise and perform smoothing operations. Finally, the camera calibration is achieved using Zhang’s method to obtain camera parameters.

The second stage is stereo matching. We employ the semi-global block matching algorithm to find corresponding disparity values between the left and right images. In order to further reduce errors and noise, we apply the weighted least squares method to process the disparity map. The weighted least squares method corrects the disparity calculation results by introducing pixel reliability weights, resulting in a smoother disparity map.

The third stage is 3D reconstruction. With the disparity map obtained from stereo matching and camera parameters, we perform triangulation calculations to convert each matching point into its corresponding 3D coordinate in the space.

### 2.1. Image Preprocessing

Thermal infrared cameras typically have a significant amount of noise in some scenes compared to visible light cameras due to effects such as low resolution of the imaging device and thermal balance between the infrared target and the background. Infrared imaging is not as desirable as expected.

To generate improved scene images and ensure high-quality disparity maps, preprocessing of thermal infrared images becomes essential. The primary purpose of preprocessing is to achieve image smoothing, noise removal, and the elimination of unnecessary details, resulting in clearer and more explicit images that facilitate subsequent stereo matching operations. Common image preprocessing methods include box filtering, mean filtering, Gaussian filtering, and others. However, traditional linear filtering methods have certain drawbacks; they cannot adaptively adjust the convolution kernel size to fit different noise environments, leading to inconsistent results. Additionally, the processing of pixels at the image edges by traditional linear filtering may cause edge blurring, which is undesirable in tasks that require edge preservation.

To overcome these challenges, researchers have turned to the use of nonlinear methods for image preprocessing. One such method is non-local mean denoising (NLM) [23], which relies on image self-similarity. NLM employs the similarity between the pixel to be processed and the surrounding pixels as weights, resulting in a weighted average of the surrounding pixels as the output value. The specific principles and steps of this approach are outlined as follows:

(1) Select a reference window: For each pixel to be filtered, a reference window is chosen, encompassing neighboring pixels around the target pixel. This reference window will be utilized to compute the filtered value of the pixel.
(2) Calculate similarity weights: For each pixel to be filtered, similarity weights are computed between the target pixel and other pixels within the reference window. The similarity can be evaluated by measuring the Euclidean distance or grayscale difference between pixels. Pixels with smaller distances or smaller grayscale differences will receive higher similarity weights. One common method for similarity calculation is the Gaussian weighted function, computed as follows:
  (1) $$\omega \left(i,j\right)=\frac{1}{C\left(i\right)}e^{-\frac{{∥v\left(N_{i}\right)-v\left(N_{j}\right)∥}_{2}^{2},\alpha }{h^{2}}}$$
  where $\omega \left(i,j\right)$ denotes the similarity between i and j. $v\left(N_{i}\right)$ and $v\left(N_{j}\right)$ are the pixel values of each pixel point within the baseline and similar blocks, respectively.${∥v\left(N_{i}\right)-v\left(N_{j}\right)∥}_{2}^{2}$ is the Euclidean distance between the two-pixel blocks, and α is the standard deviation of the Gaussian kernel function. $C\left(i\right)$ is the normalization factor, and h is the filter coefficient.
(3) Calculate the filtered value: for each pixel v, the denoised pixel value $NL\left(v\right)\left(i\right)$ is obtained by calculating the weighted average in its set of similar pixels. The formula for $NL\left(v\right)\left(i\right)$ is as follows:
  (2) $$NL\left(v\right)\left(i\right)=\sum_{j\in I}w\left(i,j\right)v\left(j\right)$$
  where $NL\left(v\right)\left(i\right)$ is the noise-reduced image pixel and $v\left(j\right)$ is the noisy image pixel. I represents the pixels in the set of similar pixels N(v).
(4) Reconstructing the image: the original pixel points are reconstructed using the pixel values obtained from non-local averaging until all pixel points of the image have been updated.

### 2.2. Camera Calibration

Camera calibration is an indispensable step in stereo matching, and its accuracy directly impacts the generation of disparity maps. The significance of camera calibration lies in accurately describing the mathematical model of camera imaging by determining both the internal and external parameters of the camera. Camera internal parameters pertain to the camera’s inherent characteristics, such as focal length, optical center, and more. On the other hand, camera external parameters encompass information about the camera’s position and orientation, ensuring seamless image fusion and collaboration among multiple cameras within machine vision systems [24]. In this paper, considering the specific imaging characteristics of infrared thermal cameras, we have devised a chessboard calibration board capable of producing significant temperature differences. This chessboard calibration board is instrumental in calibrating infrared thermal cameras for enhanced accuracy and performance.

**Figure 2:**
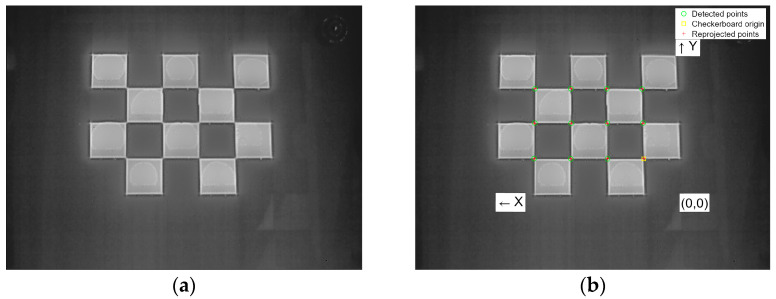
(**a**) Thermal infrared image of checkerboard calibration board, and (**b**) extraction of the calibration board.

Figure 2a displays the thermal infrared image of the calibration board, while Figure 2b demonstrates the successful extraction of corner points from the calibration board. The chessboard calibration board is fabricated using thermal insulation and ceramic heating pads. When the calibration board is energized, the temperature of the heating pads rises and is able to create a significant temperature difference from the unheated area. As depicted in Figure 2b, the homemade calibration board proves highly effective in extracting corner points, satisfactorily fulfilling the camera calibration requirements.

### 2.3. SGBM Algorithm

The SGBM algorithm is a semi-global stereo matching algorithm that conducts 2D global optimization by constraining one-dimensional paths in multiple directions, ensuring high efficiency while obtaining high-quality disparity images [25]. It primarily involves four steps: preprocessing, cost computation, dynamic planning, and post-processing.

(1) Preprocessing:

During this step, the image is processed using the horizontal Sobel operator to obtain the gradient information formula of the image. The purpose of preprocessing is to extract crucial gradient information required for cost calculation. The preprocessing formula is as follows:

(3) $$\begin{array}{c} \mathrm{Sobel}\left(x,y\right)=2\left[P\left(x+1,y\right)-P\left(x-1,y\right)\right]+P\left(x+1,y-1\right)\\ -P\left(x-1,y-1\right)+P\left(x+1,y+1\right)-P\left(x-1,y+1\right) \end{array}$$

Each pixel on the image processed by the horizontal Sobel operator is mapped to a new image, and the mapping equation is as follows:

(4) $$P_{\mathrm{new}}=\begin{array}{c} \left\{\begin{array}{c} 0,\;P<-preFilterCap\\ P+preFilterCap,\;-preFilterCap<P<preFilterCap\\ 2preFilterCap,\;P\geq preFilterCap \end{array}\right. \end{array}$$

(2) Cost Calculation

This part comprises two costs: the gradient image cost and the original image cost. Both images undergo SAD (sum of absolute differences) cost computation using a sampling-based method. SAD represents the sum of absolute differences in pixel intensities within the neighborhood of the pixel to be matched. Given that the pixel position in the left image is (x, y) and in the right image is (x+d, y), where d is the disparity value, the SAD formula is as follows:

(5) $$\;C\left(x,y,d\right)=\sum_{i=-n}^{n}\sum_{j=-n}^{n}\;\left|L\left(x+i,y+j\right)-R\left(x+d+i,y+j\right)\right|$$

where L(x+i, y+j) denotes the pixel intensity at position (x+i, y+j) in the left image, and R(x+d+i, y+j) denotes the pixel intensity at position (x+d+i, y+j)  in the right image. The parameter n represents the size of the matching window.

(3) Dynamic Planning

The planning formula is shown below:

(6) $$L_{r}\left(p,d\right)=C\left(p,d\right)+min\left\{\begin{array}{c} L_{r}\left(p-r\right),d\\ L_{r}\left(p-r,d-1\right)+P_{1}\\ L_{r}\left(p-r,d+1\right)+P_{1}\\ \underset{i}{min}L_{r}\left(p-r,i\right)+P_{2} \end{array}-\underset{k}{min}\left(p-r,k\right)\right.$$

where $P_{1}$ and $P_{2}$ represent the penalty coefficients which penalize different disparity values respectively, often ensuring $P_{1}P_{2}$, with the aim of obtaining a smooth disparity map.

(4) Post-processing

The results are further optimized through sub-pixel interpolation, left-right consistency detection, and connected regions detection. Based on these optimizations, the disparity map is processed using the weighted least squares method to enhance the accuracy of the disparity. The weighted least squares filter [26] is an edge-preserving filter designed to approximate the filtered results to the original image while smoothing the regions with lower gradients and preserving the strong gradient edges as much as possible. Given the original image as g, the spatial position of the pixel as p, the filtering result to be solved as u, and $a_{x}$ and $a_{y}$ as the weight matrices of gradients in the x and y directions, respectively, the loss function f(u) can be expressed as follows:

(7) $$\;\;\;f\left(u\right)=\sum_{p}\left({\left(u_{p}-g_{p}\right)}^{2}+\lambda \left(a_{x,p}\left(g\right){\left(\frac{\partial u}{\partial x}\right)}_{p}^{2}+a_{y,p}\left(g\right){\left(\frac{\partial u}{\partial y}\right)}_{p}^{2}\right)\right)$$

The weights represent the similarity between two pixels and the weight function is defined as follows:

(8) $$\;a_{x,p}\left(g\right)={\left({\left|\frac{\partial l}{\partial x}\left(p\right)\right|}^{\alpha }+\varepsilon \right)}^{-1}\;\;a_{y,p}\left(g\right)={\left({\left|\frac{\partial l}{\partial y}\left(p\right)\right|}^{\alpha }+\varepsilon \right)}^{-1}$$

where α is used to control the degree of contribution of the gradient to the smoothing weights, and the default value is 1.2. ε is generally taken as 0.0001, which ensures that the denominator is not zero, and avoids computational errors.

After stereo matching, using the weighted least squares filter to process the disparity map can make the disparity map more continuous, eliminate unnecessary discrete points, effectively further reduce the influence of noise, and improve the quality of the disparity map, which is very important for the subsequent 3D reconstruction.

### 2.4. Principle of Three-Dimensional Reconstruction

**Figure 3:**
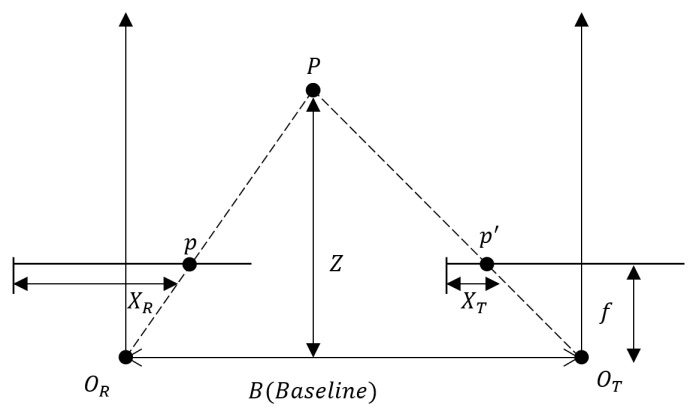
Binocular Vision Model.

The binocular stereo vision system aims to simulate the human eye’s function by employing two cameras. It calculates the three-dimensional coordinates of spatial points using the image point coordinates of the same target on the image plane captured by both cameras. The model below depicts the ideal state of a binocular stereo vision system, assuming perfect alignment between the two cameras. The cameras capture objects from different angles, and the distance between the object and the cameras is then calculated using the principle of triangulation [27]. Let $O_{R}$ and $O_{T}$ represent the optical centers of the two cameras, respectively. P is a point on the object to be measured, and its imaging points on the two camera photoreceptors are denoted as p and $p^{\prime }$, respectively. The focal length of the cameras is represented by f, B stands for the baseline between the two cameras, and Z indicates the depth information that needs to be determined. The relationship is expressed in Figure 3.

From the similarity triangle $∆Ppp^{\prime }$ with $∆PO_{R}O_{T}$, it follows that:

(9) $$\;\frac{B-\left(X_{R}-X_{T}\right)}{B}=\frac{Z-f}{Z}$$

(10) $$Z=\frac{f\times B}{X_{R}-X_{T}}$$

where $X_{R}-X_{T}$ is the disparity. Using the disparity and combining the camera parameters, we can obtain the 3D coordinates of the object in the 3D space and get the 3D point cloud map.

## 3. Results

### 3.1. Image Preprocessing Experiments

**Figure 4:**
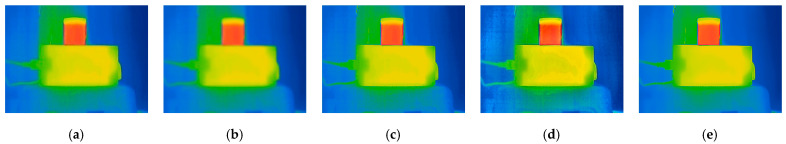
Results of image preprocessing: (**a**) original figure; (**b**) mean value filter; (**c**) Gaussian filter; (**d**) CLAHE; and (**e**) NLM.

The image preprocessing experiments encompassed the use of mean filtering, Gaussian filtering, constrained contrast adaptive histogram equalization (CLAHE), and non-local mean (NLM) algorithms to process the captured images. To evaluate the performance of these algorithms, quantitative metrics were selected and divided into full-reference evaluation metrics and no-reference evaluation metrics. The full-reference evaluation metrics include mean squared error (MSE), peak signal-to-noise ratio (PSNR), and structural similarity index (SSIM), which are used to compare the differences between the processed images and the original images. The no-reference evaluation metrics include perceptual image quality evaluator (PIQE) [28] and pixel average, which are used to assess the impact of image processing algorithms on human visual perception. The comparison of the preprocessing effects is shown in the Figure 4, while the evaluation metrics of image quality for each algorithm are presented in the Table 1. These metrics serve as crucial indicators in assessing the effectiveness and efficiency of the different image preprocessing methods.

The experimental results demonstrate that the NLM algorithm outperforms other image processing methods. Specifically, for the evaluation metrics MSE and PSNR, the NLM algorithm exhibits outstanding performance. MSE and PSNR are commonly used to assess the pixel-level difference between the original image and the image after noise reduction. The NLM algorithm excels in balancing image smoothing and preserving detail information, resulting in reduced differences between pixel values without introducing new noise. The NLM-filtered-and-processed image closely resembles the original image, as indicated by its high structural similarity index (SSIM) value of 0.93. This indicates that NLM filtering effectively reduces the noise level while retaining image details, resulting in a clearer and more realistic representation of the image. The CLAHE algorithm performs well in evaluating the PIQE metric, followed closely by the NLM algorithm. However, the relatively high pixel averages of CLAHE suggest that the algorithm may introduce some distortion or over-enhancement during the image enhancement process. Although the NLM algorithm may be relatively slow in terms of time performance, it exhibits excellent image processing results, making it a worthwhile algorithm to choose in scenes that require higher quality image reconstruction and enhancement.

### 3.2. Camera Calibration Experiment

**Figure 5:**
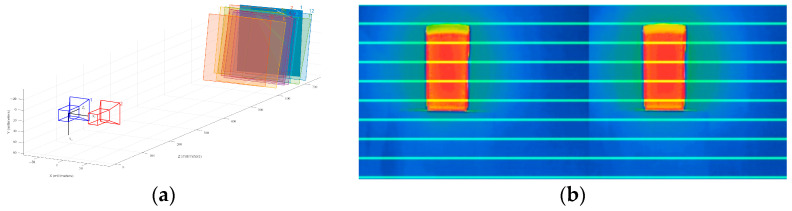
(**a**) Camera pose diagram and (**b**) calibration results.

The thermal imager detector used in the experiment is an uncooled focal plane detector with a wavelength range of 8–14 μm. The array size is 384 × 288, with a pixel size of 17 μm. The temperature measurement range is −20–120 °C, meeting the requirements of the experiment. The experiment employed MATLAB to perform the calibration of Zhang’s camera calibration method. Fifteen images of the chessboard were captured from various angles. The grid of corner points was extracted from these images, enabling the calculation of calibration parameters and completing the stereo calibration of the chessboard image. The Figure 5 presents the camera attitude map and the results of stereo correction. Additionally, the Table 2 exhibits the camera parameters obtained from the calibration process. These parameters are essential for ensuring accurate and reliable camera positioning and image reconstruction.

The experiments show that the stereo correction left and right images complete the row alignment operation, and the horizontal lines have the same corresponding positions in the left and right images with a better correction effect, which provides a stable and reliable basis for the subsequent stereo matching.

### 3.3. Stereo Matching Experiment

**Figure 6:**
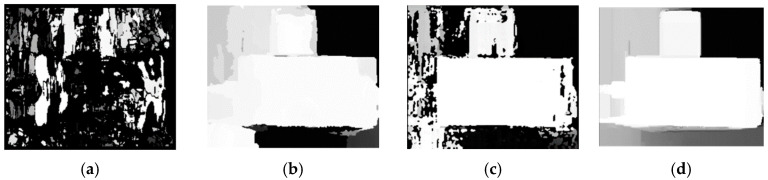
Results for stereo matching: (**a**) BM; (**b**) AD-Census; (**c**) SGBM; and (**d**) proposed algorithm.

In order to validate the proposed stereo matching algorithm, the experiment compares this paper’s algorithm with BM, AD-Census, and SGBM algorithms. The Figure 6 illustrates the running results of various stereo matching algorithms.

From the experimental results, it becomes evident that the BM algorithm lacks clear object contours, containing numerous hole areas and error matches. The AD-Census and SGBM algorithms manage to obtain the basic contour of the object, but the AD-Census algorithm exhibits error matches in the edge regions. On the other hand, the SGBM algorithm performs better than the AD-Census algorithm in terms of edge preservation but still encounters issues with hole areas. In contrast, the algorithm proposed in this paper has a clear outline of the object, and the hole areas and error matching are further reduced. In terms of time efficiency, the BM algorithm is the fastest, with a stereo matching time of only 0.002 s. In comparison, the AD-Census algorithm and the SGBM algorithm require 1.84 s and 0.005 s, respectively. Although the proposed algorithm in this paper has a runtime of 0.014 s, slightly higher than the SGBM algorithm, it can still be considered efficient. Overall, the proposed algorithm demonstrates excellent performance in stereo matching tasks.

In order to further verify the feasibility and accuracy of the algorithms, experiments were conducted by placing the targets at different locations within the distance binocular system for ranging purposes. The depth information of the targets was calculated using the results obtained from various stereo matching algorithms and then compared with the actual distance measurements to analyze the average absolute error. The Table 3 presents the ranging results of various algorithms.

Based on the experimental results, it has been observed that the algorithm proposed in this paper exhibits significant improvements in terms of measurement accuracy, showcasing commendable performance. Within the effective measurement range, the average error values for the BM, AD-Census, SGBM algorithms, and this paper’s algorithm are 30.74 mm, 34.3 mm, 28.5 mm, and 17.6 mm, respectively. Compared to the traditional SGBM algorithm, this paper’s algorithm achieves a 1.07% reduction in the absolute value of the average error. Furthermore, the average error value is lower by 1.32% and 1.66% when compared to the BM algorithm and the AD-Census algorithm, respectively. These findings highlight the superior performance of this paper’s algorithm, demonstrating its potential for enhanced accuracy in the measurement process.

### 3.4. Three-Dimensional Reconstruction

**Figure 7:**
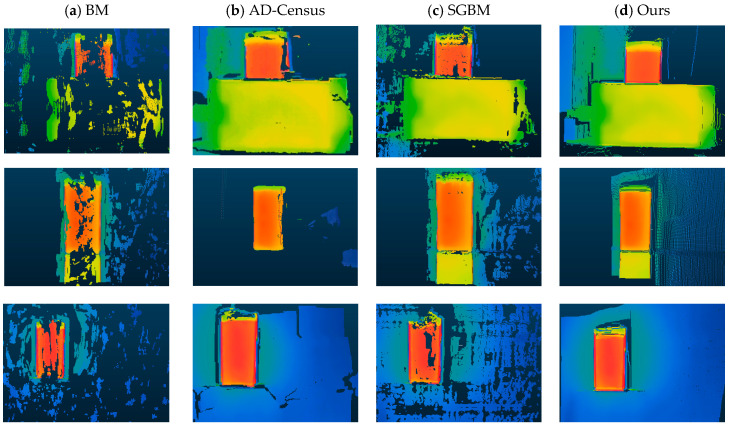
3D Reconstruction Results.

To validate the feasibility of the algorithms, experiments were conducted using three different scenes for 3D reconstruction. The disparity information obtained from the stereo matching algorithm was utilized to calculate the three-dimensional coordinates of the target points in space. Subsequently, this information was used to generate point cloud data for visualization. The reconstruction results of the BM, AD-Census, and SGBM algorithms were compared with the algorithm proposed in this paper. The Figure 7 below illustrates the 3D reconstruction results obtained using these various algorithms.

The results indicate that objects with a significant temperature difference from the environment are easier to capture, resulting in better 3D reconstruction. For objects with small temperature differences, it is difficult to find obvious feature points, which may affect the reconstruction results to some extent. Among them, the BM algorithm has the worst reconstruction effect, while the AD-Census and SGBM algorithms achieve a certain level of completion in the 3D reconstruction of objects, but the reconstruction completeness is not high. In contrast, the algorithm proposed in this paper outperforms other algorithms and demonstrates good visual effects, but it still has some shortcomings in the case of small temperature difference or reflection.

## 4. Discussion

The research focuses on thermal infrared 3D reconstruction technology based on binocular vision. It introduces a novel stereo matching algorithm that utilizes weighted least squares to address the limitations of traditional stereo matching on thermal infrared images. The proposed algorithm exhibits better matching effects and superior stereo reconstruction quality for thermal infrared images. The process begins with preprocessing the thermal infrared image using a nonlocal mean noise reduction algorithm, effectively reducing noise interference. Subsequently, the weighted least squares method is employed for semi-global stereo matching optimization, resulting in more accurate depth map estimation. Comparative analysis against traditional stereo matching algorithms reveals that the proposed algorithm significantly reduces average errors by 13.14 mm, 16.7 mm, and 10.9 mm when compared to the BM, AD-Census, and SGBM algorithms, respectively. However, the algorithm still has limitations when dealing with complex scenarios such as low thermal contrast and reflections. To overcome these challenges, future research can consider integrating other sensor data for multi-modal fusion or using more sophisticated algorithms to handle special cases.

Overall, this study presents an innovative approach to improving traditional thermal infrared image stereo matching algorithms and demonstrates exceptional results in 3D reconstruction. It has the potential to be used in fields such as firefighting, autonomous driving, and security.

## Figures and Tables

**Table 1:** Image evaluation parameters.

Algorithm Name	MSE	PSNR	SSIM	PIQE	Pixel Average	Runtime/s
mean value filter	129.01	27.02	0.86	91.67	106.81	0.059
Gaussian filter	32.8	32.97	0.95	87.69	106.88	0.012
CLAHE	208.76	24.93	0.76	57.63	112.48	0.111
NLM	11.82	37.40	0.93	83.19	106.85	0.109

**Table 2:** Binocular calibration results.

	Left Camera	Right Camera
Focal Length ($f_{x},\;f_{y}$)	(785.05, 795.83)	(793.03, 798.74)
Principal Point (u, v)	(212.29, 127.00)	(232.94, 124.39)
Rotation Matrix	$\left[\begin{array}{c} \begin{array}{ccc} 0.9997 & -0.0008 & 0.0225\\ 0.0007 & 1.0000 & -0.0025\\ -0.0225 & 0.0025 & 0.9997 \end{array} \end{array}\right]$	
Translation Vector	$\left[-59.3119,0.0486,-5.6672\right]$	

**Table 3:** Measurement result.

	Actual Distance/(mm)	BM/(mm)	AD-Census/(mm)	SGBM/(mm)	Ours/(mm)
1	900	856.9	947.7	860.8	861.2
2	950	915.4	972.3	971.7	954.9
3	1000	972.3	1042.5	976.9	986.3
4	1050	1025.4	1078.5	1019.9	1033.0
5	1100	1123.7	1130.5	1014.4	1086.0

## Data Availability

The data underlying the results presented in this paper, which were collected in H.L. Laboratory, are not publicly available at this time but may be obtained from the corresponding author upon reasonable request.
